# Effects of naturally-arising HIV Nef mutations on cytotoxic T lymphocyte recognition and Nef's functionality in primary macrophages

**DOI:** 10.1186/1742-4690-8-50

**Published:** 2011-06-22

**Authors:** Philip Mwimanzi, Zafrul Hasan, Ranya Hassan, Shinya Suzu, Masafumi Takiguchi, Takamasa Ueno

**Affiliations:** 1 Center for AIDS Research, Kumamoto University, Kumamoto, Japan

## Abstract

**Background:**

Although HIV can infect several cellular subsets, such as CD4^+ ^T lymphocytes and macrophages, it remains unclear whether an HIV infection in macrophages supports cytotoxic T lymphocyte (CTL) escape. Here, we tested two naturally-arising mutations located in the well-conserved polyproline region of Nef for their effects on CTL recognition, Nef's functionality, and viral replication capacity in macrophages. These mutations were selected because they are known to cause CTL escape in the context of T lymphocytes.

**Findings:**

Monocyte-derived macrophages (MDMs) infected with the wild-type virus, but not with variant viruses, were efficiently killed by CTL clones targeting Nef epitopes, VY8 (VPLRPMTY) and RY11 (RPQVPLRPMTY). The CTL-escape mutation, Arg^75^Thr, or Arg^75^Thr/Tyr^85^Phe double mutation, reduced the HLA class I down-regulation activity and, interestingly, increased the susceptibility of virus-infected MDMs to recognition by CTLs targeting a different epitope. The same mutations reduced the CCR5, but not CD4, down-regulation activity. Moreover, the Nef variants were impaired for Hck activation and enhancement of viral replication in MDMs.

**Conclusions:**

These results suggest that HIV-infected MDMs are killed by CTLs targeting Nef epitopes, contributing to selection and adaptation of CTL-escape viral variants.

## Findings

Several different cellular subsets such as CD4^+ ^T lymphocytes, macrophages, and dendritic cells can be targets for an HIV infection; although they differentially support HIV replication and persistence *in vivo *[1-3]. Macrophages may be the early target of HIV, but are highly resistant to the cytopathic effects of an HIV infection and continuously produce infectious virions for a long period of time [4,5]. It is thought that the differences in fitness of viral replication among the different cellular environments could influence the selection and adaptation of viral quasispecies in these cells. The HLA class I-restricted CD8^+ ^cytotoxic T lymphocyte (CTL) response is thought to play an important role in controlling HIV replication [6-8] and to mediate a major selective force for the emergence of viral variants [9,10]. Certain CTL escape mutations, in well-conserved regions of Gag and Nef, have been reported to impose functional constraints on these proteins and to modulate viral replication in the context of T lymphocytes [11-13]. However, in the context of macrophages, the selection of CTL escape variants and functional adaptation of viral proteins are not yet fully understood. We previously showed that the HLA-B35-restricted CTL responses toward a well-conserved proline-rich region in Nef results in the emergence of a CTL escape mutation, either Arg75Thr or Tyr85Phe, from phylogenetically different viral quasispecies even within an HIV-infected host [13]. These mutations constrain some of the important Nef functions in CD4^+ ^T cells [13]. Here we tested whether an HIV-1 infection in macrophages would have any influence on CTL recognition and escape as well as Nef's functionality and adaptation in the infected macrophages.

### Susceptibility of HIV-infected macrophages to recognition by the cognate CTLs

We previously reported that in HIV-infected patients with HLA-B35, the Nef protein elicits dominant CD8 T cell responses [14], with the short epitope VY8 (Nef_78-85_; VPLRPMTY) being the early epitope, which subsequently shifts to the amino terminal-extended longer epitope RY11 (Nef_75-85_; RPQVPLRPMTY) [13]. Autologous virus sequence analysis revealed that the mutations Tyr85 to Phe (85F) and Arg75 to Thr (75T) are associated with the early and chronic phase of an HIV infection, respectively, in HIV-infected individuals with *HLA-B35 *but that these 85F and 75T mutations are derived from phylogenetically different lineages [13].

**Figure 1:**
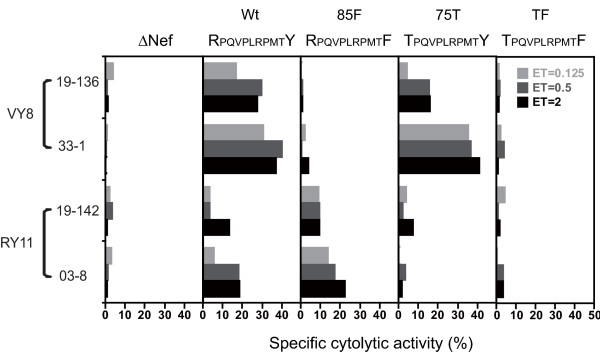
**Susceptibility of HIV-infected MDMs to recognition by the cognate CTLs**. Cytotoxic activity of HLA-B35-restricted CTL clones specific for VY8 (VPLRPMTY) and RY11 (RPQVPLRPMTY) epitopes in Nef toward HIV-infected MDMs is shown. MDMs were isolated from an HIV-negative donor (HLA-B^*^35:01^+^) and infected with wild-type or one of the variant viruses indicated. The resultant HIV-infected MDMs (2 × 10^3^/well) were then mixed with CTL clones at various effector-to-target cell ratios (E/T) for 6 hr at 37°C after having been labeled with ^51^Cr. The frequency of HIV-infected cells among target cells as determined by intracellular p24 Ag expression was 48.7, 55.4, 51.0, and 48.8% for wt, 85F, 75T, and TF variants, respectively. CTL 19-136 and 19-142 were derived from the same HIV-infected donor (019), and CTL 33-1 and 03-8 were derived from different donors, 033 and 03, respectively. CTL activity toward uninfected cells was deducted from the data as background. An additional experiment showed similar results.

We first examined CTL activity toward macrophages infected with HIV-1 strain JRFL, in which *nef *gene had been replaced with that of strain SF2 (referred as JRFL-SF2nef) and its variants. In this JRFL-SF2nef, we had created unique restriction sites, *Cla *I and *Not *I adjacent to the ends of the *nef *open reading frame [15] and confirmed that the resultant viruses, prepared by transfecting 293 T cells with JRFL and JRFL-SF2nef, had comparable replication capacity in primary monocyte-derived macrophages (MDMs) (data not shown). To prepare mature MDMs, CD14^+ ^cells were isolated from PBMCs of HIV-negative donors, in accordance with the human experimentation guidelines of Kumamoto University, and cultured for 7 days in the presence of 100 ng/ml of macrophage colony-stimulating factor (Peprotech GmbH, Germany). Previously established CTLs, specific for VY8 and RY11, [13,14] were highly cytotoxic toward MDMs infected with wild-type (wt) HIV-1, suggesting that HIV-infected MDMs were a preferable target for CTLs. The VY8-specific CTLs showed higher cytotoxicity toward wt virus-infected MDMs than did the RY11-specific CTLs (Figure 1), in good agreement with the observation obtained with HIV-infected CD4^+ ^T cells [13,16]. In contrast, VY8- and RY11-specific CTLs failed to kill primary MDMs infected with 85F and 75T viruses, respectively (Figure 1), indicating that the 85F and 75T single mutations conferred escape from CTLs specific for VY8 and RY11, respectively, but not simultaneously. In contrast, the TF virus could escape from both types of CTLs (Figure 1). It should be noted that Western blot analysis of Nef proteins in virus-producing cells showed a comparable level of Nef expression among wt and all variant viruses except for ΔNef (data not shown).

### Effects of the Nef mutations on Hck activation

**Figure 2:**
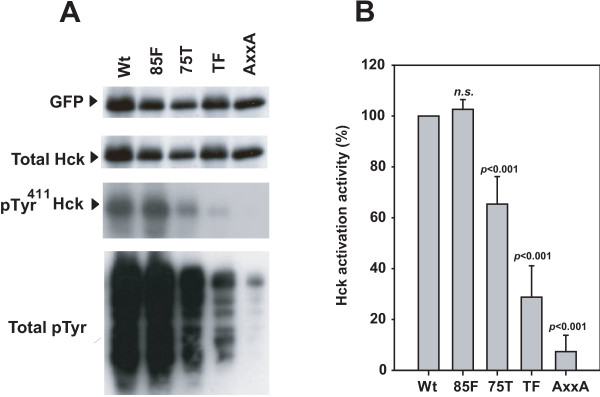
**Hck activation**. (A) HEK293 cells were transfected with cDNA encoding GFP alone or Nef-GFP fusion proteins in the presence of Hck plasmid, and analyzed by Western blotting with anti-GFP (FL; Santa Cruz), anti-Hck (clone 18; Transduction Laboratories, Lexington, KY), anti-Hck phosphorylated at tyrosine 411 (Hck-pTyr411; Santa Cruz) and anti-phosphotyrosine (PY99; Santa Cruz) as indicated. The Nef variants tested are indicated in the figure. A representative datum set of 3 independent experiments is shown. (B) Quantification of Hck activation by Nef. The indicated values represent the Hck activation activity after the level of phosphorylated Hck had been normalized to the amounts of total Hck. The values presented were calculated from the data shown in panel A, and are relative to the wild-type control arbitrarily set to 100%. Data represent the means ± SD of 3 independent experiments, and statistical analysis was performed based on ANOVA with multiple comparisons *vs. *wt (Bonferroni *t*-test). *n.s*., not significant.

Nef is known to associate via its PxxP motif with the SH3 domain of several different cellular kinases including Hck [17,18]. We tested whether the CTL-escape variants in the PxxP region would affect the Hck activation by Nef by using the *in vitro *Hck activation assay as described earlier [19] (Figure 2A). Expectedly, the wild-type Nef showed robust Hck activation; whereas the AxxA variant Nef (Pro76Ala and Pro79Ala) did not show substantial activation (Figure 2B). The 85F variant Nef did not affect Hck activation, whereas the Hck activation was substantially reduced by the 75T and TF variants of Nef (Figure 2B). These results suggest that CTL-escape variants in the PxxP motif affect Hck activation in macrophages.

### Effects of the Nef mutations on HLA class I down-regulation

**Figure 3:**
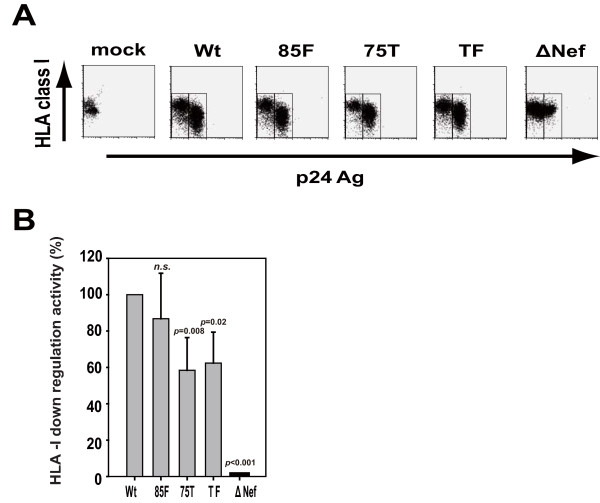
**HLA class I down-regulation in HIV-infected MDMs**. (A) MDMs prepared from an HIV-negative donor were infected with wild-type or variant viruses as indicated. Cells were stained with 7-amino-actinomycin D (7-AAD; BD Biosciences, CA) and anti-HLA class I allotype antibody SFR8-B6 followed by intracellular staining with FITC-labeled anti-p24 Gag mAb (KC-57; Beckman Coulter, CA) as described before [13]. In flow cytometric analysis (FACS Canto II, BD Biosciences), cells negative for 7-AAD were gated and analyzed for their fluorescence intensity for HLA class I and p24 Gag. (B) The same experiment as above was done by using 3 additional HIV-negative donors. The HLA class I allotype-specific antibodies used were either SFR8-B6 or A11,1 M as appropriate for each donor. The relative down-regulation activity of HLA class I by wt Nef and its variants is presented relative to that of the wild-type Nef activity set to 100%. Data represent the means ± SD of all 4 donors, and statistical analysis was performed based on ANOVA with multiple comparisons *vs. *wt (Bonferroni *t*-test). *n.s*., not significant.

Because Nef helps HIV-infected cells to evade CTL lysis by down-modulating cell-surface HLA-I and the PxxP motif is critical for this activity [13,20,21], we examined the HLA-I down-regulation activity by Nef in MDMs infected with wt and variant viruses by flow cytometry (Figure 3A). The surface levels of HLA-I within p24^+ ^subsets in wt virus-infected MDMs were much reduced compared with those in uninfected cells (Figure 3B) and that no HLA-I down-regulation was observed in ΔNef virus-infected MDMs (Figure 3B). In contrast, both the 75T and the TF variant viruses showed substantially diminished down-regulation activity; whereas the 85F variant virus showed down-regulation activity comparable to that of the wt (Figure 3B).

### Susceptibility of HIV-infected MDMs to recognition by CTLs of another specificity

**Figure 4:**
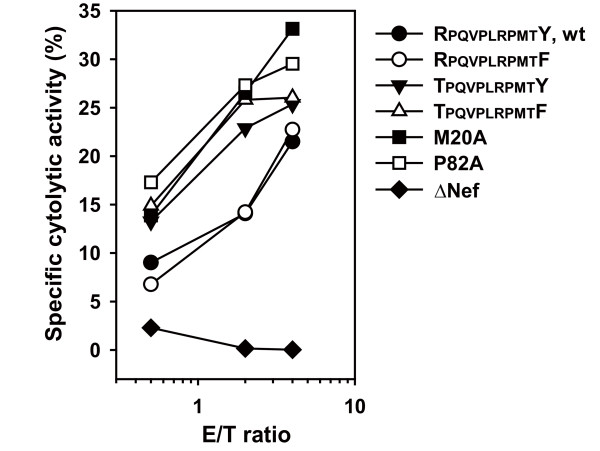
**Susceptibility of HIV-infected MDMs to recognition by CTLs of another specificity**. MDMs prepared from an HIV-negative donor (HLA-A^*^24:02^+^) infected with the indicated viruses were used as target cells for cytolysis by an HLA-A24-restricted CTL clone specific for the Nef epitope (Nef_138-147_: RYPLTFGWCF). The frequency of HIV-infected cells among the target cells, as determined by intracellular p24 Ag expression, was 41.4, 48.3, 44.5, 40.8, 40.0, and 45.0% for wt, 85F, 75T, TF, M20A, and P82A variants, respectively. CTL activity toward uninfected cells was deducted from the data as background. An additional experiment showed similar results.

We postulated that the impaired Nef-mediated down-regulation activity of HLA-I in MDMs could influence the susceptibility to killing of HIV-infected MDMs by CTLs. To test this, we first created the variant virus having M20A or P82A (numbering based on the SF2 strain) because these mutations have been shown to completely disrupt the Nef-mediated HLA-I down-regulation activity [22,23]. We then assessed the cytolytic activity of CTL clones specific for another Nef epitope presented by HLA-A24 (Nef_138-147_: RYPLTFGWCF) toward MDMs infected with wt, M20A, or P82A viruses. Although the amino-acid sequences in the epitope region of A24-Nef were the same among the wt and these variant viruses tested, the CTL-mediated killing activity toward MDMs infected with M20A and P82A variant viruses was much increased compared to those infected with the wt virus (Figure 4).

Next, we also determined CTL cytotoxic activity toward MDMs infected with 75T, 85F, and TF variant viruses. The A24-Nef CTLs showed the most potent activity toward MDMs infected with either the 75T or TF variant viruses; whereas their cytotoxic activity was less potent toward MDMs infected with either the wt or the 85F mutant virus (Figure 4). These data suggest that the diminished HLA-I down-regulation (i.e., increased level of cell-surface HLA-I) in MDMs infected with the 75T and the TF mutant viruses (Figure 3) resulted in increased susceptibility to killing by CTLs of another specificity (Figure 4), leading to a possible selective disadvantage for the variant viruses under anti-HIV CTL responses.

### Effects of the Nef mutations on down-regulation of viral receptors

**Figure 5:**
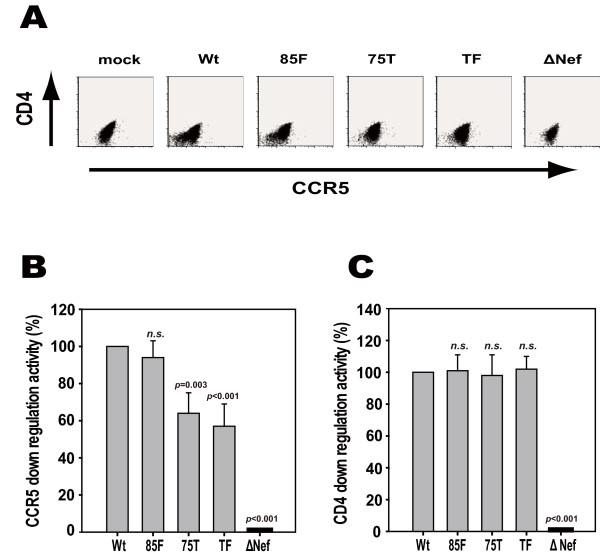
**Viral receptor down-regulation in HIV-infected MDMs**. (A) MDMs prepared from an HIV-negative donor were infected with wt or variant viruses as indicated. The cells were stained with 7-AAD and allophycocyanin-Cy7 anti-human CD4 mAb (Biolegend, CA) and phycoerythrin-Cy7-conjugated anti-human CCR5 mAb (BD Biosciences) followed by intracellular staining with antibody against p24 Gag. In flow cytometric analysis, cells negative for 7-AAD and positive for p24 Gag were gated and analyzed for their fluorescence intensity for CD4 and CCR5. (B, C) The same experiment as above was done by using 3 additional HIV-negative donors. The relative down-regulation activity of wt Nef and its various variants toward CCR5 (panel B) and CD4 (panel C) is presented, with the wt Nef activity set to 100%. Data represent the means ± SD of all 4 donors, and statistical analysis was performed based on ANOVA with multiple comparisons *vs. *wt (Bonferroni *t*-test). *n.s*., not significant.

We also examined whether Nef's down-regulation activity of viral receptors, i.e., CD4 and CCR5, could be influenced by the mutations in HIV-infected MDMs (Figure 5A). The cell-surface expression of CCR5 was substantially reduced in wt virus-infected MDMs but not affected in the ΔNef variant virus-infected ones (Figure 5B). Interestingly, the 85F variant virus showed CCR5 down-regulation activity comparable to that of the wt virus; whereas the 75T and TF variant were substantially impaired in this activity in MDMs (Figure 5B). In contrast, CD4 down-regulation activity was not affected for all of the viruses with mutated Nefs except for ΔNef (Figure 5C), consistent with the observation that CD4 down-regulation activity is mediated by a specific region in Nef other than the PxxP motif [21].

### Effects of the Nef mutations on viral replication

**Figure 6:**
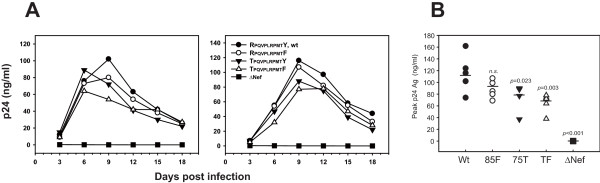
**HIV replication in MDMs**. (A) MDMs prepared from 2 HIV-negative donors were incubated at 3 × 10^5 ^cells/well in a 96-flat bottomed well plate, infected for 6 hr with wt or variant HIV-1 at 10 ng of p24 antigens, and then continuously cultured at 37°C in fresh culture medium for an additional 18 days. Culture supernatants were collected and replaced with fresh medium every 3 days. To monitor viral replication, the concentration of p24 Gag in the culture supernatant was quantified by ELISA for their p24 Gag concentration (ZeptoMetrix Corp. Buffalo, NY). (B) The same experiment was done by using MDMs prepared from 3 additional HIV-negative donors. The peak p24 Ag values were plotted and statistically analyzed based on ANOVA with multiple comparisons *vs. *wt (Bonferroni *t*-test). *n.s*., not significant. Horizontal bars represent the means of data obtained from all 5 donors

We finally examined whether the mutations would differently affect the enhancement of viral replication in MDMs. In MDMs from 2 HIV-negative donors, the wt HIV-1 showed the highest replication among the various viruses tested; whereas the ΔNef variant showed much decreased replication (Figure 6A), consistent with the previous observation [24]. The replication of the 85F variant virus was partially impaired in MDMs from one of the donors and was comparable to that of the wt virus in MDMs from the other donor (Figure 6A). In contrast, the replication of the 75T and TF variant viruses was impaired in MDMs from both donors (Figure 6A). To account for this donor variability, we summarized the results from a total of 5 donors in Figure 6B. Because the peak of the virus replication was between 6 to 12 days after infection, depending on the donor and the virus, the peak p24 Ag values of each of the viruses are presented and were used for statistical analysis (Figure 6B). The 75T and the TF variant viruses showed significantly diminished capacity for viral replication compared with the wt; whereas the 85F virus did not show much difference in replication capacity (Figure 6B).

## Discussion and Conclusions

Although the Nef protein is thought to have very high mutational plasticity, we showed here that the naturally-arising CTL escape variants in the well-conserved PxxP region in Nef alone or in combination can modulate some pathogenic functions of Nef in the context of human primary macrophages infected with a CCR5-tropic virus. There are 2 different aspects of CTL-mediated functional constraints on the PxxP-dependent Nef activities in MDMs reported here, one through immune evasion activity (HLA-I down-regulation activity) and the other acting on the intrinsic capacity to boost viral replication and persistence (Hck activation, viral co-receptor down-regulation activity, and enhancement of viral replication). In particular, one of the single mutants, 75T, impaired these Nef activities in MDMs infected with a CCR5-tropic virus. This is in line with the previous report showing that 75T mutation modulated Nef-stimulated viral replication in immature dendritic cell/T cell cocultures infected with a CCR5-tropic virus [25] although this mutation alone had virtually no influence on the same Nef activities in primary CD4^+ ^T cells infected with a CXCR4-tropic virus in the previous study [13]. In addition, the 75T mutation, located outside the VY8 epitope, reduced the cytolytic activity of VY8-specific CTLs in the context of CD4^+ ^T cells [13], but did not affect their cytolytic activity in the context of MDMs (Figure 1), suggesting the differential intracellular processing of the VY8 peptide between CD4^+ ^T cells and MDMs. This observation is in line with the previous report showing a substantial difference in intracellular processing of antigenic HIV peptides between monocytes and lymphocytes [26]. Overall, these results suggest that an antigenic variation of viruses can differentially influence viral replication and persistence between cellular subsets because of their different effects on the intracellular antigen-processing machinery, the susceptibility to CTL killing, as well as the fitness cost to viral replication.

Of particular interest are the data showing that the CTL-escape Nef mutation, 75T, impaired HLA-I down-regulation activity by Nef and rendered the HIV-infected MDMs more susceptible to killing by CTLs with another specificity. Such phenomenon was also observed in the context of CD4^+ ^T cells in our previous study [13]. However, wt-virus-infected cells, regardless of CD4^+ ^T cells or MDMs, could be killed to some extent by CTLs, suggesting that the Nef-mediated HLA-I down-regulation is insufficient for HIV to escape from CTL recognition and that, CTL-escape variant viruses are selected and emerged. Conversely, Swigut *et al.*, [27] reported that monkeys infected with SIV containing *nef *mutations that selectively eliminated MHC down-regulation activity exhibited higher level of SIV-specific CD8 T cell responses. In any event, an important question remains to be addressed which is how significant is Nef-mediated HLA-I down-regulation activity for HIV replication and persistence in HIV-infected humans.

Although HLA-B*35-restricted CTLs targeting PxxP region of Nef can impose functional constraints in viral replication in this study, we did not find any beneficial effects on clinical parameters (such as CD4 count and viral load) in HIV-infected patients with HLA-B*35 as well as those with HLA-B*35 and HLA-A*24 in our cohort to date (data not shown). Functional impairment in Nef induced by CTL-escape variants could be compensated later by mutations at secondary sites in Nef. For example, an inverse dose-response relationship has been observed between the number of CTL-escape mutations in Nef and CD4 counts in patients in a large population study [28]. Therefore, only some CTL-escape variants may play a role in modulating Nef functions *in vivo*, such as in the case of HLA-B57^+ ^elite suppressors [29]. Further studies using a large number of clinically-isolated *nef *alleles are needed to extend this observation, such as how Nef-specific CTL responses, Nef functions, and clinical outcome of HIV-infected individuals are related to each other at the population level.

## Competing interests

The authors declare that they have no competing interests.

## Authors' contributions

PM, MT, and TU designed the study. PM, ZH, RH, SS, and TU conducted the experiments. PM, SS, and TU wrote the paper. All authors read and approved the final manuscript.
